# Leaf Counting: Fusing Network Components for Improved Accuracy

**DOI:** 10.3389/fpls.2021.575751

**Published:** 2021-06-10

**Authors:** Guy Farjon, Yotam Itzhaky, Faina Khoroshevsky, Aharon Bar-Hillel

**Affiliations:** Department of Industrial Engineering and Management, Ben-Gurion University of the Negev, Be'er Sheva, Israel

**Keywords:** image-based plant phenotyping, leaf counting, counting with convolutional neural networks, fusing network components for counting, growth rate estimation

## Abstract

Leaf counting in potted plants is an important building block for estimating their health status and growth rate and has obtained increasing attention from the visual phenotyping community in recent years. Two novel deep learning approaches for visual leaf counting tasks are proposed, evaluated, and compared in this study. The first method performs counting *via* direct regression but using multiple image representation resolutions to attend leaves of multiple scales. The leaf count from multiple resolutions is fused using a novel technique to get the final count. The second method is detection with a regression model that counts the leaves after locating leaf center points and aggregating them. The algorithms are evaluated on the Leaf Counting Challenge (LCC) dataset of the Computer Vision Problems in Plant Phenotyping (CVPPP) conference 2017, and a new larger dataset of banana leaves. Experimental results show that both methods outperform previous CVPPP LCC challenge winners, based on the challenge evaluation metrics, and place this study as the state of the art in leaf counting. The detection with regression method is found to be preferable for larger datasets when the center-dot annotation is available, and it also enables leaf center localization with a 0.94 average precision. When such annotations are not available, the multiple scale regression model is a good option.

## 1. Introduction

Object counting is important for a variety of tasks in the agriculture and phenotyping domains. Estimating the number of fruits, flowers, and trees helps farmers make better decisions on cultivation practices, plant disease prevention, and the size of the harvest labor force (Rahnemoonfar and Sheppard, 2017). For example, estimating the number of flowers in a field can be used to predict harvest dates from peak flowering, track the response of flowering to environmental conditions, and evaluate the effects of cultural practices (Adamsen et al., 2000). The chemical thinning process of apple trees starts by counting the number of flowers during the blossom period, for flowering level and peak day estimation (Farjon et al., 2019). Determination of wheat grains quality is done by counting wheat seedlings in the field and estimating their density (Liu et al., 2016).

In this study, we focus on the task of leaf counting. The number of leaves a plant has is one of the visual key traits (phenotype) describing its development and growth (Dobrescu et al., 2017). It enables growth rate estimation and is related to the health status of the plant and its yield potential (Telfer et al., 1997; Walter and Schurr, 1999). Manually measuring the traits of the visual plant is a slow, tedious, and expensive process (Jiang et al., 2019), and it usually requires the presence of specialized investigators (Giuffrida et al., 2016). Hence, these traits are measured on a small random sample of plants, which might lead to a measurement bias (Aich and Stavness, 2017). An accurate automated leaf counting system will enable faster measurements that will decrease costs, and improve the overall accuracy of per-plant estimations (Aich and Stavness, 2017). Due to the importance of this task, a Leaf Counting Challenge (LCC) was initiated in recent years in the Computer Vision Problems in Plant Phenotyping (CVPPP) community and public benchmarks (Scharr et al., 2014; Bell and Dee, 2016; Minervini et al., 2016) are available.

This study presents two network architectures for leaf counting, one based on direct regression and the other on combining detection with regression. For direct regression, we suggest improving upon existing art by explicitly employing fusion over multiple-scale analysis. The number of leaves is regressed from multiple image resolutions, created by a Feature Pyramid Network (FPN) (Xie et al., 2015), thus accounting for both small and large leaves. For each scale, a network is trained to provide both an estimation of the leaves count and the variance of the estimation. The multiple estimates obtained from the different image resolutions are then fused based on their estimated variance. This method is termed Multiple-Scale Regression (MSR). We examine several techniques for fusing the estimators, including min-variance selection and a Maximum Likelihood Estimation (MLE) solution.

The second proposed method treats counting as a two-stage process, where leaf detection is done prior to count regression, yet both are done in a single end-to-end network. Following the density estimation literature (Xie et al., 2015, 2018), we develop an algorithm employing “leaf center” point annotations during training. At several stages, the detection network is regressing a “heat map,” a map with Gaussians of pre-defined parameters placed upon the annotated leaf centers (Lempitsky and Zisserman, 2010). The heat map is gradually refined to obtain more exact leaf positions, thus allowing better separation between small adjacent leaves. An initial count estimate is obtained by applying Non-Maxima Suppression (NMS) and global summing layers. This detection-based estimate is fused with additional features to provide the final count estimate *via* direct regression. This method is termed Detection with Regression Network (DRN). The training of the network is driven by two losses accounting for the detection and count regression accuracy.

**Figure 1:**
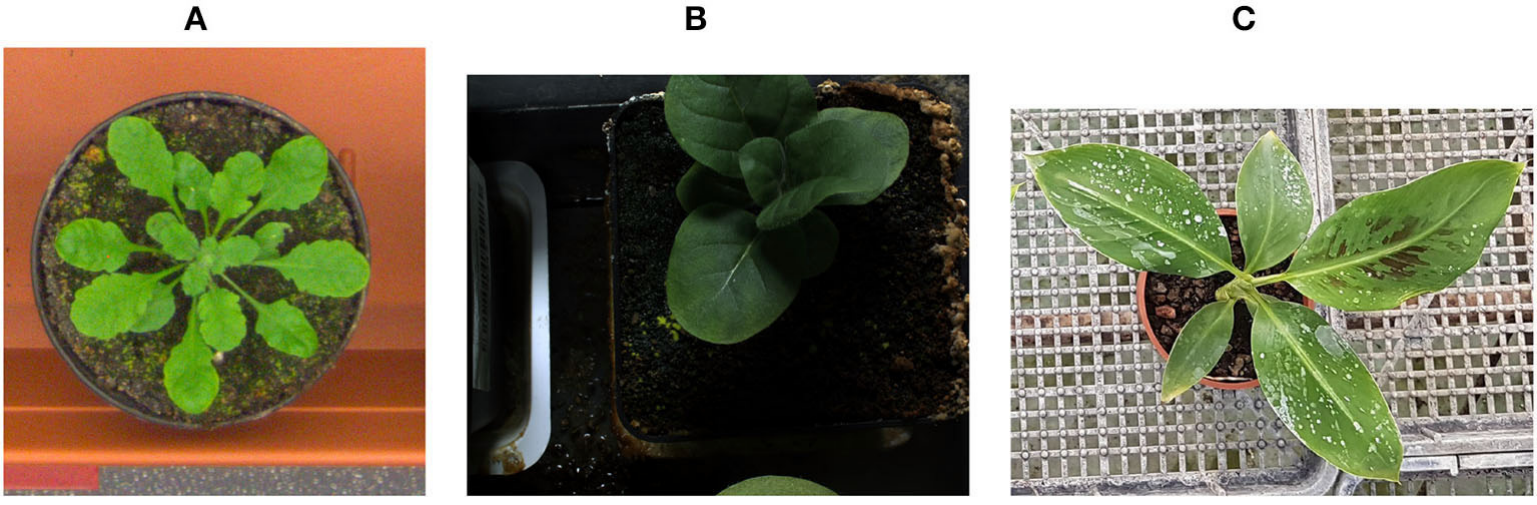
Data example of three different plants used in the experiments. **(A)** Rosette plant, **(B)** Tobacco plant, **(C)** Banana plant.

The suggested counting methods were tested on the publicly available LCC datasets containing tobacco and *Arabidopsis* plants (Bell and Dee, 2016; Minervini et al., 2016), as well as on a larger dataset of images of the banana plants. Examples of images from the datasets can be seen in Figure 1. Both methods obtained improvement over the current state-of-the-art on the LCC datasets, evident by the results obtained on a hidden test set held by the dataset owners. While for small datasets, the methods are comparable in performance, and DRN is preferable when a sufficient sample size is available. This method, which also provides detection of the individual location of the leaves, is hence currently the leading leaf counting method to the best of our knowledge.

The main contributions of this study are:

- It shows that merging ideas from detection and direct regression methods in a single network provides improved accuracy (compared to any other current method) in the leaf counting task. Specifically, a detection refinement mechanism is proposed based on repeated heat map regression with decreasing Gaussian kernel size.
- It shows that for direct regression, using estimators from multiple image scales is beneficial, but that it requires careful fusion of the estimators based on additional variance estimation.
- The methods suggested provide a new state-of-the-art in the leaf counting from RGB images task, as measured on the LCC challenge.

The entire code is freely accessible at https://github.com/farjon/Leaf-Counting. This work is an extension of a previously presented conference paper (Itzhaky et al., 2018), which was supplemented in several important ways. First, the DRN was improved by employing successive refinement of the heat maps produced with Gaussian kernels of decreasing size. Second and more important, careful experiments were conducted to understand the role of data size on the LCC datasets and using a novel larger dataset with 1,016 images of banana plants. These experiments reveal that the DRN method is superior to the MSR for a large enough dataset, a fact which was not clear enough in the conference paper version. Finally, a comprehensive comparison to existing art is provided, showing that the suggested algorithms compare favorably with the best current methods.

## 2. Background

With the growing need for systematic plant phenotyping (Großkinsky et al., 2015) and the development of recent Convolutional Neural Network (CNN)-based techniques (Ren et al., 2015; He et al., 2016), visual leaf counting has attracted considerable attention (Giuffrida et al., 2016; Dobrescu et al., 2017; Lu et al., 2017; Teimouri et al., 2018; Jiang et al., 2019; Kuznichov et al., 2019). A basic but appealing idea is to perform counting by using some standard detection or segmentation network architecture (Ren et al., 2015; Redmon et al., 2016; He et al., 2017; Lin et al., 2017b) to detect the leaves. In this way, one also obtains information additional to counting, the exact image locations of the leaves. This information maybe valuable for applications requiring further processing, like detecting diseases or examining the morphological structure of the leaf. In Romera-Paredes and Torr (2016) a segmentation-based approach was developed and tested on the LCC dataset. First, the image goes through a Fully Convolutional Network (FCN) providing a high-dimensional feature map representation. This map is then used as input for a Recurrent Neural Network (RNN) model in which each layer application segments a different leaf. The leaf count is the number of segmented objects. The study of Ren and Zemel (2017) uses the same techniques as in Romera-Paredes and Torr (2016), but in addition, offers the ability of instance segmentation. A recent segmentation and counting network was presented in Kuznichov et al. (2019), where new algorithms were suggested for the generation of synthetic plant images. These images are then used as data augmentation for network training. The resulting network provides the state-of-the-art results in the Leaf Segmentation Challenge (LSC),^1^ which is a segmentation plus counting benchmark on the same datasets (Scharr et al., 2014; Bell and Dee, 2016; Minervini et al., 2016) as the LCC. However, such segmentation-based methods have a significant drawback for counting since only successfully segmented leaves are counted, and their results in the counting evaluation metric are less competitive.

More direct approaches to counting use annotations requiring less effort, like dot annotations, marking only the object center (Arteta et al., 2016), or even just image-level counts (Dobrescu et al., 2017). When dot annotations are available, counting is often approached *via* a density estimation approach (Xie et al., 2015, 2018). These methods are successful for approximate counting of large object quantities (hundreds or thousands, like crowds in stadiums, cars in large parking lots, etc.), but are less suited for accurate counting of small quantities as required in the leaf counting task. If only image-level counts are available for training, counting tasks can be addressed by a direct regression approach, where a regressor is learning a direct function from image to count estimation (Dobrescu et al., 2017; Lu et al., 2017). The natural regressor choice is an adapted deep CNN. The advantages of this approach are the simplicity of the algorithm, hence the ability to train it from a smaller sample size, and the alleviation of the annotation burden. The currently leading results reported on the leaf counting CVPPP challenge datasets were obtained with a direct regression approach (Dobrescu et al., 2017; Teimouri et al., 2018).

Considering such direct approaches, Giuffrida et al. (2016) proposed a flat (non-deep) learning-based approach for leaf counting in rosette plants. They used a supervised regression model, applied to image-based descriptors learned in an unsupervised manner. Teimouri et al. (2018) treat leaf counting as a classification task, where each possible count results in a limited range is a different label. They use an ensemble of 20 similar models based on Inception-v3 architecture (Szegedy et al., 2016), pre-trained on ImageNet (Deng et al., 2009), and fine-tuned on the leaf counting datasets. They report good results but their evaluation is partial, with 168 images randomly sampled from the five LCC datasets (A1–A5). Therefore, the results cannot be fully compared to LCC bench-marked results. In Aich and Stavness (2017) the authors treated the counting task as a two-staged task. First, they used SegNet (Badrinarayanan et al., 2017) to isolate the plant from the background. Then, they used the segmented mask and the RGB image as the input to a VGG-16 based regressor. Though Aich and Stavness (2017) reports better results than Giuffrida et al. (2016), they are inferior to those of Dobrescu et al. (2017) in which a regression model based on the ResNet-50 architecture (He et al., 2016) was suggested. Trained on multiple leaf datasets and using data augmentation, they obtained the winning results in the LCC event of 2017, and the best LCC benchmark results prior to this study. The model uses only the leaf count annotations while training. Giuffrida et al. (2018) shows identical results on the LCC 2017 dataset as in Dobrescu et al. (2017), but by using multi-modal data (including near infrared channel and a fluorescence channel, in addition to RGB images), the authors showed state-of-the-art results on Cruz et al. (2016) dataset. However, when multi-modal data is not available, the methods proposed in this study are with superior accuracy.

## 3. Methods

### 3.1. Image Datasets

Two datasets were used in the experiments:

- Leaf Counting Challenge data: The challenge includes four different datasets termed A1–A4. Datasets A1, A2, and A4 contain images of the *Arabidopsis* plant and the A3 dataset contains images of young tobacco plants. Datasets A1, A2, and A3 are relatively small containing 128, 31, and 27 images, respectively, while A4 is larger with 624 images. Images were taken from a top viewpoint, using a camera positioned ~1 m above the plants, under controlled illumination conditions. Each image contains a single plant, which was monitored across several weeks (up to 7 weeks for some plants). Hence, plants age varies between several days and several weeks. For testing, the LCC organizers also provided the A5 dataset, which is sampled from A1 to A4 test sets. This dataset was created to test if models generalize well across datasets and are not dataset-specific. In addition, a combined dataset was created, which includes all images from A1 to A4, and was termed Ac (A5 is a subset of Ac). More information is shown in Scharr et al. (2014), Bell and Dee (2016), and Minervini et al. (2016).
- Banana leaves (BL) data: This dataset is larger than the LCC datasets, containing 1,016 images from a top viewpoint collected at plantations in the north of Israel. This dataset is different from the LCC dataset mainly since the leaves are relatively large and elliptical. The banana leaves were annotated using the *via* annotation tool (Dutta and Zisserman, 2019).

Each image was labeled in two annotation levels. The first is the number of leaves, and the second is a leaf center dot annotation. The size of the images varies between 441 × 441 and 2, 448 × 2, 048, and they were resized to 800 × *W* where *W* was chosen to keep the original aspect ratio. To enrich the datasets, random transformations were applied including rotation, scaling, vertical and horizontal flips.

### 3.2. Direct Regression Architecture and Design

Direct regression algorithms are widely used for various counting tasks (Aich and Stavness, 2017; Dobrescu et al., 2017; Rahnemoonfar and Sheppard, 2017; Ubbens and Stavness, 2017; Aich et al., 2018). For example (Dobrescu et al., 2017), the winner of LCC 2017, uses the ResNet-50 architecture as a regressor. The top classification layers of the network were removed and replaced with a regression “head,” containing two fully connected layers followed by a single leaf estimate output neuron. We aim to improve upon Dobrescu et al. (2017) by considering estimation at multiple scales. The regressor used is based on an FPN architecture (Lin et al., 2017a), extending a backbone network to produce multiple resolutions of representation. As in Lin et al. (2017a), a backbone of ResNet-50 (He et al., 2016) is used, trained on ImageNet data (Deng et al., 2009).

#### 3.2.1. An FPN for Multiple Scale Representation

**Figure 2:**
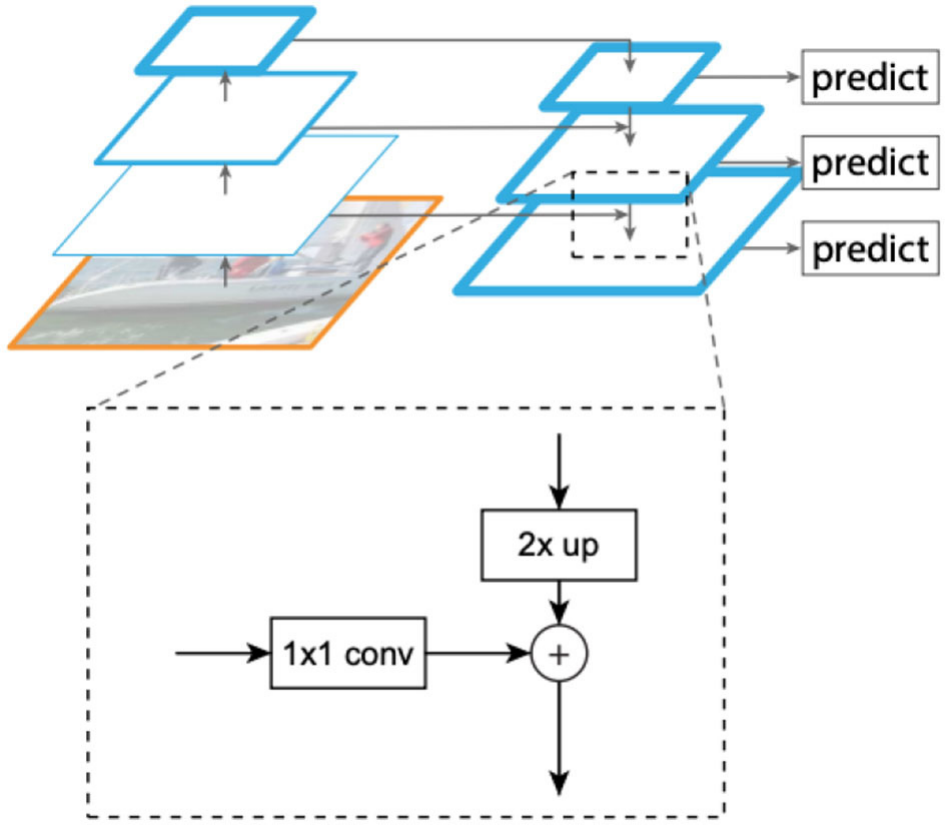
Creating representations at multiple resolutions from a standard Convolutional Neural Network (CNN) (Lin et al., 2017a). Each such tensor (termed *P*_2_ − *P*_7_) involves a *X*2 up-sampling of the representation at the higher level (to get the rich semantics), and adding information from a corresponding lower CNN level through 1 × 1 convolutions (to add better spatial resolution). The produced multiple scale representations are close to being “equivalent” in the sense that a single classifier or regressor can be trained and applied to all of them.

The input to the FPN is an image of arbitrary size, and the output is a feature pyramid of proportionally sized tensor representations at multiple levels. The FPN architecture aims to provide a rich representation with semantic features typically available at higher layers of a CNN, yet at multiple spatial resolutions. To obtain this, tensor representations of increasing spatial resolutions are created iteratively starting from the highest level representation. A schematic view of this process is shown in Figure 2. The pyramid used by the method includes representation tensors denoted by *P*_3_−*P*_7_ in Lin et al. (2017a), where pyramid level *P*_*j*_ has a resolution of 2^*j*^ lower than the input. All pyramid levels have *C* = 256 channels. The details of the pyramid generally follow Lin et al. (2017a) with a few modest differences, based on Lin et al. (2017b). Specifically, we include the higher and more semantic pyramid levels *P*_6_ and *P*_7_ instead of *P*_2_, which provides better localization (less important for counting). Like Lin et al. (2017b), *P*_6_ is obtained *via* a 3 × 3 stride-2 convolution on ResNet-50 layer *C*_5_, and *P*_7_ is computed by applying Rectified Linear Unit (ReLU) followed by a 3 × 3 stride-2 convolution on *P*_6_. A regression sub-model is then applied at each scale.

#### 3.2.2. Count Regression Sub-model

**Figure 3:**
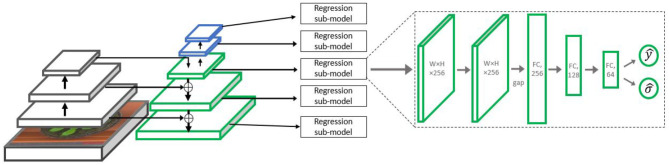
The Multiple-Scale Regression (MSR) architecture. The left side contains the backbone ResNet-50 network, followed by the Feature Pyramid Network (FPN). On the right, the regression sub-model is displayed. Such a sub-model is attached to each *P*_*j*_ level of the FPN,with *j* = 3, . . . , 7.

The regression sub-model includes two 3 × 3 ReLU convolution layers with 256 output maps, followed by a global average pooling operation, flattening the maps to a compact 256 × 1 representation. This vector is fed into two fully connected layers with decreasing sizes of 128 and 64, respectively, followed by the output layer. This layer contains two output neurons which are estimates of the mean and variance of the expected number of leaves (as shown in details below). The architecture is summarized in Figure 3. As in the RetinaNet (Lin et al., 2017b) detection architecture, all the regressor sub-models share their weights, so effectively the same regressor is applied to each pyramid level. We hence use five different image pyramid levels for gaining five different leaf count estimators, with their fusion described in the section “fusing the predictors of the sub-models.”

#### 3.2.3. Modeling the Uncertainty of Each Sub-model

Following Kendall and Gal (2017), the regression modules have two outputs, ŷ and ${\hat{\sigma }}^{2}$, corresponding to the expected leaf count and its variance. The training data is a labeled sample of images ${\left\{I_{i},y_{i}\right\}}_{i=1}^{N}$ with *y*_*i*_ being the leaf count label. We train the network to minimize the following loss:

(1) $$\begin{array}{l} L_{r}=\overset{N}{\underset{i=1}{\sum }}\frac{1}{2}\exp\left(-s_{i}\right)\cdot ||\hat{y_{i}}-y_{i}{||}^{2}+\frac{1}{2}s_{i} \end{array}$$

where $s_{i}=\log{\hat{\sigma_{i}}}^{2}$, and $\hat{y_{i}}=ŷ\left(I_{i}\right)$, $\hat{\sigma_{i}}=\hat{\sigma }\left(I_{i}\right)$ are the outputs of the regressor over image *i*. This loss is applied independently to the five scales and summed to get the total loss minimized. Note that the loss is the log of a Gaussian density (up to an additive constant), but with the mean and variance being input-dependent estimates. This means that in image space regions where the regression is not accurate (i.e., where large deviations occur between ŷ and the true *y*, a larger ${\hat{\sigma }}^{2}$ is inferred to reduce the loss. The opposite happens in areas of accurate prediction.

#### 3.2.4. Fusing the Predictions of the Sub-models

The output of the count regression model is the prediction of the mean and the variance for each of the scales. Given count and variance estimates for the scales of the five FPN, new fusion options beyond plain averaging become possible. Keeping an input image index *i* fixed (and hence omitted), denote the multiple estimators for this image by ${\left({ŷ}^{j},{\hat{\sigma }}^{j}\right)}_{j=1}^{J}$. One intuitive way would be to choose the model with the lowest degree of uncertainty, i.e., the one with the lowest predicted variance for the specific input image:

(2) $$\begin{array}{l} ŷ=\hat{y^{k}}\;with\;k=argmin\left(\hat{\sigma^{j}}\right) \end{array}$$

While this choice is intuitive, we know that the MLE for the consolidation of Gaussian distributions is different. Specifically, the MLE has the closed-form solution:

(3) $$\begin{array}{l} ŷ=\frac{\overset{J}{\underset{j=1}{\sum }}\frac{1}{{\left(\hat{\sigma^{j}}\right)}^{2}}\hat{y^{j}}}{\overset{J}{\underset{j=1}{\sum }}\frac{1}{{\left(\hat{\sigma^{j}}\right)}^{2}}} \end{array}$$

It should be noted that the second method assumes observation independence between the results of the sub-models. This assumption is not fulfilled, since the sub-models receive as input similar representation at different resolutions. However, in practice, the fusion method given by Equation (3) was performed better and was used as default in the experiments.

### 3.3. Detection-Based Counting

In this approach, the network is taught to first detect leaf centers, using dot annotation provided at training, and only then count them. Toward this task, a two-dimensional heat map was created for every training image to serve as an intermediate output goal. In the heat map, a two-dimensional Gaussian is placed around each leaf center (as shown in detection sub-model section regarding Gaussian size). Each Gaussian was normalized such that its peak pixel (i.e., the leaf center location) equals to 1. The network was trained to estimate this heat map, and then use it to regress the number of leaves. The heat map, in turn, is estimated using an iterative process in which a coarse initial heat map is gradually refined through the network stages to reach a final heat map of fine spatial resolution.

**Figure 4:**
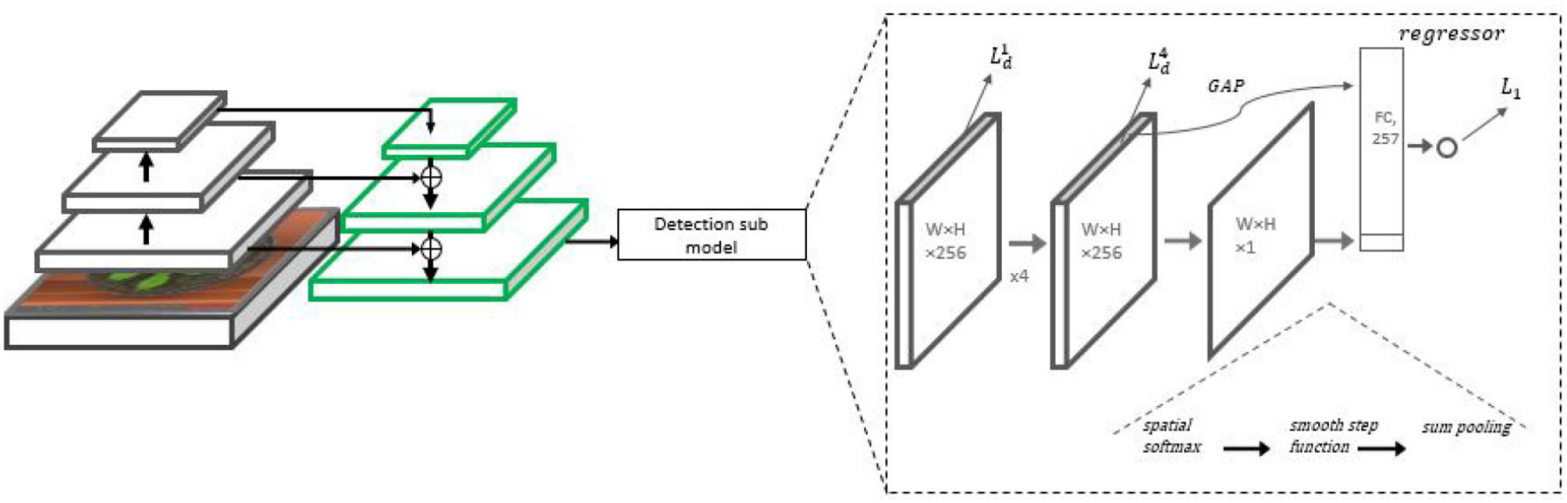
The Detection with Regression Network (DRN) architecture. On the left side, the backbone ResNet-50 with the FPN is displayed, from which *P*_3_ representation is taken for further processing. On the right, the detection sub-model is presented, including four 3 × 3 ReLU convolutional layers repeatedly regressing the leaf heat map. A counting head summarizes the final heat map estimate into an initial count estimate. The final count estimate is regressed from a combination of the initial estimate and a feature vector extracted from the final heat map.

Like in the MSR model, the initial representation used is based on the FPN architecture with a ResNet-50 backbone. The model does not employ multiple scale analysis, but the FPN is used nevertheless due to the combination of semantic features from the higher layers and the high spatial resolution obtained at its lower layers. A detection sub-model regressing the heat map is placed on top of the high-resolution pyramid scale (*P*_3_). Then, a count regression sub-network accepts the heat map and tensors leading to it as input and predicts the final count output. The architecture is summarized in Figure 4.

#### 3.3.1. Detection Sub-model

The detection sub-model is a small FCN, containing four 3 × 3 ReLU convolutional layers with 256 filters each. The final layer in the sub-model contains a single 2*D* map approximating the ground truth leaf center heat map, and it is produced by an additional 1 × 1 ReLU convolutional layer with a single filter. $\hat{p}$ and *p* denote the value of the network estimation and the target heat map, respectively, for a single map location (dropping location and layer indices for convenience). The minimized loss is a weighted smooth-L1 loss

(4) $$\begin{array}{l} L_{d}\left(\hat{p},p\right)=\{\begin{array}{ll} \left(1-w\right)\cdot Huber\left(\hat{p},p\right) & ,\text{where p > 0}\\ w\cdot Huber\left(\hat{p},p\right) & ,\text{otherwise} \end{array} \end{array}$$

With

(5) $$\begin{array}{l} Huber\left(\hat{p},p\right)=\{\begin{array}{ll} |\hat{p}-p|-\frac{1}{2} & ,\text{where}|\hat{p}-p|\geq 1\\ \frac{1}{2}{\left(\hat{p}-p\right)}^{2} & ,\text{otherwise} \end{array} \end{array}$$

The weight parameter *w* (*w* = 0.1 in the experiments) keeps the total weight of positive pixels in the heat-map high. This directs the optimization toward accurate regression of these values, rather than the pixels with the value zero, which outnumber them considerably. Although the final heat map is predicted following all four convolutional layers, each of those layers predicts a heat map independently using an additional loss term $L_{d}^{k}$ for *k* = 1, …, 4. The ground truth heat maps guiding the estimates produced by early convolutional layers were created using larger Gaussian kernels. Hence the first convolutional layers estimate a coarse heat-map (created with a 7 × 7 Gaussian kernel), in which sometimes Gaussians may overlap, while the final layer predicts a finer heat-map (created with a 3 × 3 Gaussian kernel). Each of the layers in the sub-model are guided using the same loss function as the final loss. Denoting by $p_{ij}^{k},{\hat{p}}_{ij}^{k}$ the true and predicted values at location (*i, j*) of layer *k*, the minimized detection loss is the sum of losses overall pixels and layers:

(6) $$\begin{array}{l} L_{d}=\overset{4}{\underset{k=1}{\sum }}L_{d}^{k}=\overset{4}{\underset{k=1}{\sum }}\underset{\left\{\left(i,j\right)\right\}}{\sum }L_{d}\left({\hat{p}}_{ij}^{k},p_{ij}^{k}\right) \end{array}$$

#### 3.3.2. Counting Sub-network

Given an estimated heat-map, an initial count estimate can be based on finding the number of Gaussian centers. Ideally, this number is the required leaf count. However, assuming imperfect detection, it is preferable not to use it directly as the final count, but as a strong feature provided to the final regressor. To properly count the Gaussian centers, we suggest to incorporate a smooth NMS procedure, shrinking each Gaussian to a delta function, followed by a global sum operation. Hence, the following NMS function is applied to the estimated heat map $\hat{P}\in R^{N\times M}$.

(7) $$\begin{array}{l} \;Q=MaxPool\left(\hat{P},\left(K,K\right)\right)\\ {\hat{P}}_{NMS}=\hat{P}\cdot exp\left(-\beta \cdot |\hat{P}-Q|\right) \end{array}$$

**Figure 5:**
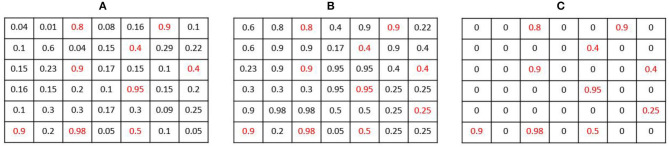
Smooth Non-Maxima Suppression (NMS) procedure example: **(A)** an estimated heat map $\hat{P}$. **(B)** The resulting map *Q* after applying the *MaxPool* operation with *K* = 2. Local maxima are highlighted in red. **(C)** the output map ${\hat{P}}_{NMS}$.

Where *MaxPool*(*X, F*) is the operation of max-pooling with stride 1 and an *F* filter size. Following this operation, a pixel in the output map ${\hat{P}}_{NMS}$ keeps a value close to its original value in $\hat{P}$ if it was the highest value of $\hat{P}$ in a *K* × *K* neighborhood (as shown in example Figure 5). The experiments indicate that large β values were effective, entailing a sharp NMS keeping only the winners. *K* is chosen to be the same value used in the heat map creation, so a single pixel is expected to remain active from a full Gaussian.

The NMS operation keeps mostly the pixels that are local maxima, but it does not remove noise, so ${\hat{P}}_{NMS}$ still contains small values in non-center pixels. We hence apply a smooth step function (sigmoid) layer of the form $y\left(x\right)=\frac{1}{1+e^{-\rho \left(x-t\right)}}$ to keep only values greater than some threshold (*t* = 0.8, ρ = 15 were used). The result is a detection map $\hat{D}$ containing the estimated leaf centers as its active pixels. A global sum pooling layer applied to $\hat{D}$ then gives a single number $C_{\hat{D}}$, the detection-based estimate for the leaf count.

Beyond the detection-based estimate $C_{\hat{D}}$, additional features are used for the final count regression. A Global Average Pooling (GAP) operation is applied to the fourth convolutional layer (the layer predicting the final heat map), obtaining a 256-dimensional feature vector *V*_4_. The final count estimator Ĉ is obtained by applying linear regression in the final layer, i.e., $Ĉ=W\cdot \left[V_{4},C_{\hat{D}}\right]$ where $\left[V_{4},C_{\hat{D}}\right]$ is a concatenated 257 features vector. We train the final counter to minimize an *L*_1_ loss estimated based on the true count, which is added to the detection loss (Equation 6).

## 4. Results and Discussion

We describe next the training procedure and evaluation metrics. Then, the results of the two suggested methods are presented and compared to current art methods. Finally, performance analysis of the suggested method is done using visualization, ablation studies, and experiments with varying sample sizes.

### 4.1. Training Procedure

Following Dobrescu et al. (2017), validation results were obtained using a 4-fold cross-validation procedure, and the average results over the folds were reported. Test results were obtained by evaluating the model on a hidden held-out set kept by the challenge organizers (Scharr et al., 2014; Bell and Dee, 2016; Minervini et al., 2016) (the model predictions were sent to the LCC organizers for evaluation). The tested model was trained over the accumulated dataset, with 75% of the data used for model fitting and 25% as a validation set for the early stopping criterion. We did not use the provided foreground plant mask in either of the models. In all experiments, ADAM (Kingma and Ba, 2014) optimizer was used with a learning rate of 10^−5^.

### 4.2. Evaluation Metrics

For count regression evaluation, we used the metrics provided by the LCC workshop organizers (as shown in Dobrescu et al., 2017). *C* and Ĉ denote the true and estimated count, respectively, and these include

- The average distance *E*[Ĉ − *C*], where *E*[·] denotes the average over test images. This is not used as an accuracy score, but it measures the bias of the predictor (its tendency to over or underestimate the count) − DiC.
- The average *L*_1_ distance *E*[|Ĉ − *C*|] − |*DiC*|.
- The fraction of accurate agreement *E*[1_Ĉ = =*C*_] − agreement [%].
- The Mean-Squared Error *E*[(Ĉ − *C*)^2^] − MSE.

For estimation of the detection performance, a recall-precision curve was used with the Average Precision (AP) metric—the area under the curve. To determine if a leaf detection (an active point in $\hat{D}$) is a hit or a miss we use the criterion introduced in the Percentage of Correct Keypoints (PCK) computation (Yang and Ramanan, 2013). With PCK, a leaf detection point is considered a hit if the distance between it and a ground truth leaf center is lower than α·*max*(*w, h*) where (*w, h*) are the width and height of the bounding boxes surrounding the entire object (plant). Like in Yang and Ramanan (2013) we used α = 0.1 to test the detection performance.

### 4.3. Results and Comparison to Previous Work

Cross-validation results on the datasets of the LCC challenges, as well as the new banana leaf dataset, are reported in Table 1. The results are more stable for datasets *A*1 and *A*4, which are larger. There is no clear winner in this examination, but the proposed MSR and DRN have a slight advantage over the LCC winner (Dobrescu et al., 2017). Also, it can be seen that when the training set is small, the MSR method has an advantage, while for larger datasets (*A*4 and *BL*) the DRN method usually performs better. In Table 2, the results of the test held-out set of the LCC are presented and compared to current best methods. The results here are more decisive. First, the MSR and DRN methods have an advantage over previous art for most datasets and metrics. Second, DRN has a clear leading position overall methods (including MSR) in all the three performance indices (|*DiC*|, agreement, and MSE). This is specifically seen in the large sample and heterogeneous conditions “all” and *A*5.

**Table 1:** Cross-validation results on separate datasets.

**Dataset**		**DiC**			**|*****DiC*****|**			**(%)**			**MSE**		
**Train**	**Test**	**MSR**	**DRN**	**Dobrescu et al. (2017)**	**MSR**	**DRN**	**Dobrescu et al. (2017)**	**MSR**	**DRN**	**Dobrescu et al. (2017)**	**MSR**	**DRN**	**Dobrescu et al. (2017)**
A1	A1	−0.06	**0.03**	−0.81	**0.53**	0.56	0.94	**58**	51	25	0.78	**0.7**	1.38
A2	A2	**0.61**	0.73	−2.38	**1.26**	2.05	2.38	**43**	22	38	**3.71**	7.82	12.9
A3	A3	**0.14**	−0.45	−0.57	**1.07**	1.6	1.43	**30**	21	14	**1.89**	3.83	2.57
A4	A4	**0.03**	−0.21	0.1	0.71	**0.6**	0.91	45	**49**	35	1.15	**0.85**	1.54
Ac	A1	**0.02**	0.21	0.28	0.66	0.6	**0.53**	44	45	**56**	0.86	**0.7**	0.72
Ac	A2	0.63	0.45	**−0.38**	1.09	**0.86**	0.88	35	**52**	38	3.33	2.44	**1.38**
Ac	A3	0.14	**0.02**	0.71	**0.71**	0.75	**0.71**	41	29	**57**	0.94	**0.83**	1.57
Ac	A4	**−0.01**	0.06	0.12	0.65	**0.63**	0.69	47	**51**	46	**0.97**	1.0	1.01
BL	BL	**0.05**	0.09	0.07	0.41	**0.37**	0.46	61	**69**	56	0.43	**0.42**	0.51

*Results are presented for the algorithms: Multiple Scale Regression (MSR), Detection and Regression Network (DRN), and direct regression of Dobrescu et al. (2017), the LCC 2017 challenge winner. The best results for each metric is presented in bold*.

**Table 2:** Results of the MSR and the DRN on the held-out test set, with comparison to the winner of the 2017 LCC (Dobrescu et al., 2017) and to Giuffrida et al. (2018) which shows identical results.

		**A1**	**A2**	**A3**	**A4**	**A5**	**All**
DiC							
	MSR	−0.27 (1.21)	**−0.22 (0.67)**	**−0.04 (1.61)**	**0.15 (1.08)**	**0.10 (1.22)**	**0.07 (1.22)**
	DRN	**−0.24 (0.94)**	−0.67 (1.00)	−0.25 (1.01)	0.26 (1.09)	0.12 (1.08)	0.09 (1.08)
	Dobrescu et al. (2017) and Giuffrida et al. (2018)	−0.39 (1.17)	−0.78 (1.64)	0.13 (1.55)	0.29 (1.10)	0.25 (1.21)	0.19 (1.24)
	Aich and Stavness (2017)	−0.33 (1.38)	−0.22 (1.86)	2.71 (4.58)	0.23 (1.44)	0.80 (2.77)	0.73 (2.72)
	Giuffrida et al. (2016)	−0.79 (1.54)	−2.44 (2.88)	−0.04 (1.93)	–	–	–
|*DiC*|							
	MSR	0.70 (1.02)	**0.44 (0.53)**	1.14 (1.12)	0.76 (0.78)	0.84 (0.90)	0.83 (0.90)
	DRN	**0.48 (0.83)**	0.67 (1.00)	**0.75 (0.72)**	**0.75 (0.83)**	**0.73 (0.80)**	**0.72 (0.81)**
	Dobrescu et al. (2017) and Giuffrida et al. (2018)	0.88 (0.86)	1.44 (1.01)	1.09 (1.10)	0.84 (0.76)	0.90 (0.85)	0.91 (0.86)
	Aich and Stavness (2017)	1.00 (1.00)	1.56 (0.88)	3.46 (4.04)	1.08 (0.97)	1.66 (2.36)	1.63 (2.30)
	Giuffrida et al. (2016)	1.27 (1.15)	2.44 (2.88)	1.36 (1.37)	–	–	–
%							
	MSR	57.6	**55.6**	32.1	42.9	41.7	42.3
	DRN	**63.6**	**55.6**	**35.7**	**43.5**	**42.6**	**43.7**
	Dobrescu et al. (2017) and Giuffrida et al. (2018)	33.3	11.1	30.4	34.5	33.2	32.9
	Aich and Stavness (2017)	30.3	11.1	7.1	29.2	23.8	24.0
	Giuffrida et al. (2016)	27.3	44.4	19.6	–	–	–
MSE							
	MSR	1.48	**0.44**	2.54	**1.19**	1.50	1.49
	DRN	**0.91**	1.33	**1.07**	1.25	**1.17**	**1.17**
	Dobrescu et al. (2017) and Giuffrida et al. (2018)	1.48	3.00	2.38	1.28	1.53	1.56
	Aich and Stavness (2017)	1.97	3.11	28.0	2.11	8.28	7.90
	Giuffrida et al. (2016)	2.91	13.33	3.68	–	–	–

*A5 dataset is a sample from A1 to A4 datasets. “All” dataset is a combination of all the datasets (A1–A4). The reported results are the mean and standard values of each metric. It is noticeable that the DRN algorithm gets a better score in almost each of the indices. The best results for each metric is presented in bold*.

### 4.4. Detection and Regression Network Analysis

**Figure 6:**
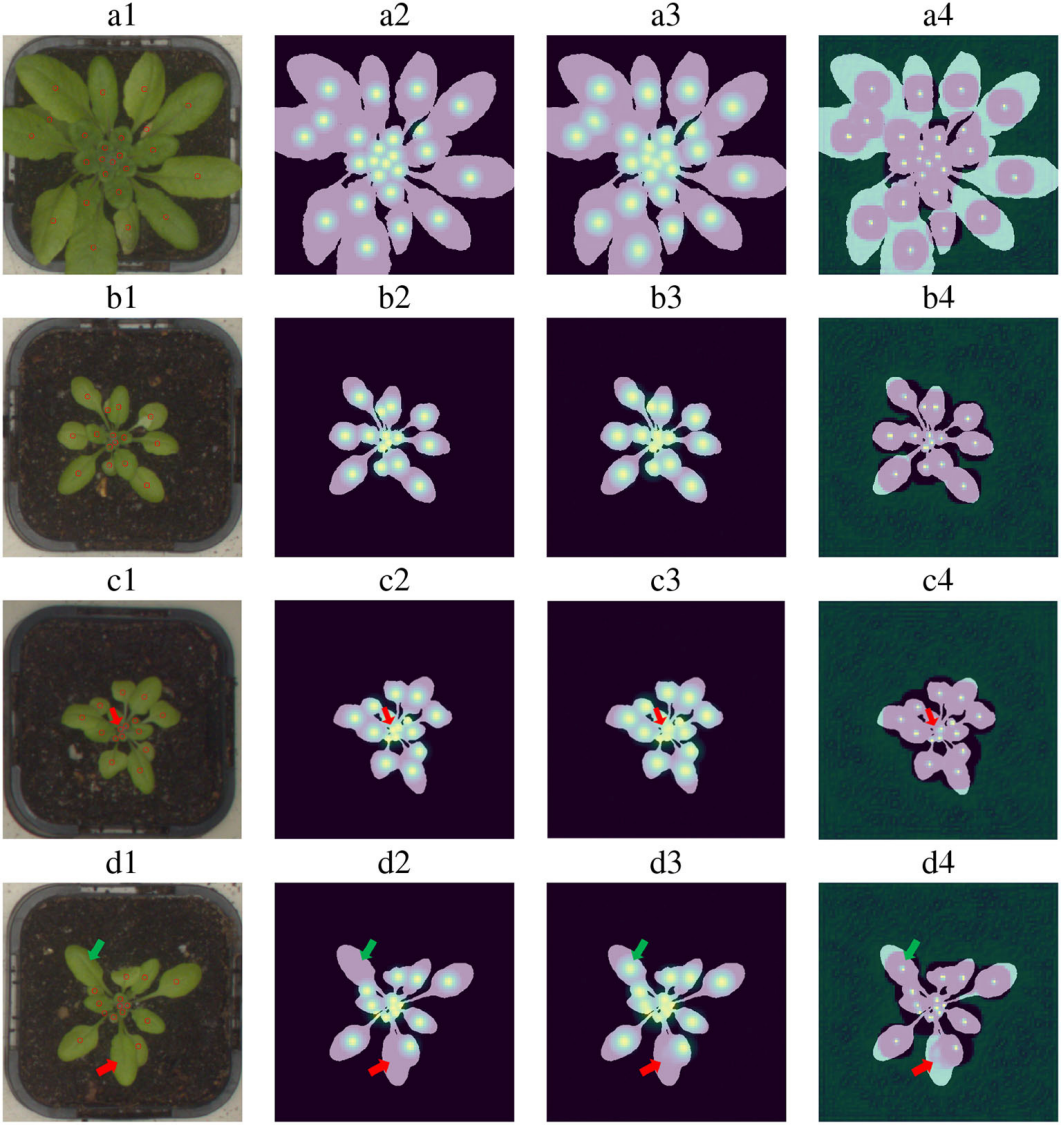
Leaf center detection examples for four images. **Column 1:** Input images with their ground truth point annotations. **Column 2:** Ground truth Gaussian heat maps generated using human annotation. **Column 3:** Gaussian heat maps inferred by the model. **Column 4:** Leaf center predictions after the non-maxima suppression operation. Examples “a” and “b” present successful detection cases. Examples “c” and “d” include miss detection examples and wrong annotation found by the model (a miss-detection is pointed with a red arrow, wrong annotation with a green arrow).

Some examples of the performance of the detectors are shown in Figure 6. As can be seen from these examples, the estimated Gaussian heat map successfully finds the vast majority of the leaves. Misses occur mostly for close leaves, high leaf occlusion cases, and very small leaves. For example, row *c* in Figure 6 shows a typical leaf miss detection. Notice that the leaf is extremely small and that its Gaussian is too close to other Gaussians.

Table 3 shows the metric scores for several ablated model versions on the largest data set of the LCC, *A*4. The results indicate that each of the examined elements contributes to the performance of the detector. Specifically, combining the detection with a final regression and the usage of multiple intermediate losses for detection is of high importance. This model obtained 0.94 AP when trained on 80% of the Ac dataset and validated on 20% of it, indicating the achievement of both high recall and high precision. For example, for a recall value of 0.7, the precision is 0.987.

**Table 3:** Ablation table for the DRN.

**Condition**	**DiC**	**|*DiC*|**	**(%)**	**MSE**
1. Full model	−0.02	0.6	49	0.85
2. Fixed GT heat maps	0.02	0.63	48	0.93
3. No data Aug.	0.1	0.71	42	1.09
4. No multiple losses	0.6	1.14	41	4.91
5. Detection only	0.78	1.36	40	7.7

*The table includes cross-validation results on the A4 dataset. 1. The full model. 2. Using fixed size kernels for generating the ground truth Gaussians of the heat maps. 3. Training without data augmentation. 4. Using a single detection loss at the end of the detection sub-model. 5. Using count estimator $C_{\hat{D}}$ as the single feature for count regression*.

### 4.5. Multiple Scale Regression Analysis

Table 4 presents the results of intermediate algorithm versions between the baseline of Dobrescu et al. (2017) and the suggested multiple-scale regression. The results are of cross-validation experiments on the A1 dataset. The baseline model following Dobrescu et al. (2017) includes a ResNet-50 backbone, global average pooling on top of the *C*_5_ convolution layer, two fully connected layers in decreasing sizes, and a final single neuron predictor. As can be seen, two main steps improve the performance over this baseline: moving to pyramid features and using the maximum likelihood technique for the fusion of the estimators.

**Table 4:** Incremental improvements over a baseline in the direct regression model, cross-validation results on A1 dataset.

**Index**	**Input**	**Loss**	**Description**	**DiC**	**|*DiC*|**	**Agreement (%)**	**MSE**
1	*C*_5_	*L*_2_*loss*	Baseline	0.15	0.62	48	0.9
2	*P*_3_	*L*_2_*loss*	pyramid 1 level	−0.07	0.55	53	0.76
3	*P*_3_−*P*_7_	*L*_2_*loss*	Avg fuse	0.18	0.66	44	0.91
4	*P*_3_−*P*_7_	*Gauss*	$min\;\hat{\sigma }$ fuse	−0.08	0.62	45	0.82
5	*P*_3_−*P*_7_	*Gauss*	MLE fuse	−0.06	0.53	58	0.78

*The table rows present the following: 1. A baseline is similar to Dobrescu et al. (2017) (the implementation of it). 2. Replacing the input of the regressor with the middle pyramid level P5. 3. Using five pyramid levels, averaging the results. 4. Using five pyramid levels with the min-sigma fusion of the predictors (Equation 2). 5. Five pyramid levels with MLE fusion (Equation 3)*.

**Figure 7:**
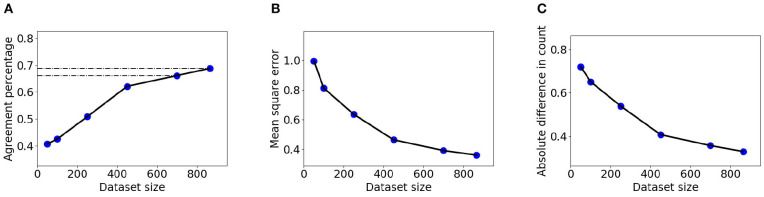
Detection and Regression Network network accuracy as a function of dataset size for BL data, measured using the LCC evaluation criteria. **(A)** Agreement index. **(B)** MSE index. **(C)** |*DiC*| index. the method earns, though with diminishing returns, from samples including many hundreds of data points.

The larger dataset BL enables measurement of the relation between sample size and counting accuracy in a larger domain than before. Figure 7 shows |*DiC*|, MSE, and counting agreement as a function of training set size. In the experiment, 150 images were held out for accuracy estimation, and models were trained using subsets of 50, 100, 250, 450, 700, and 866 images from the BL dataset. The presented results are the average of five experiments with different train-test splits. It can be seen that the DRN model significantly benefits from additional examples in the explored range.

### 4.6. Inference Speed

We implemented the model in Python, using Keras and TensorFlow framework. We used a single 1080-Ti GTX Nvidia GPU for training and testing. While training the models takes up to 24 h (on the largest dataset), the inference is fast. MSR reaches 20 FPS (0.05 s per image) and DRN reaches 7 FPS (0.14 s per image) for images of average size 450 × 460, typical for all LCC datasets except for A3. As expected, MSR is significantly faster than DRN since it uses a direct regression approach. For A3, containing larger images (2, 448 × 2, 048), inference took on average 0.34 s for MSR and 0.43 for DRN.

### 4.7. Discussion

The methods developed here have several limitations, related to difficulty factors like leaf occlusion, the number of plants in the image, illumination conditions, and leaf shape diversity. While occlusion is present in the LCC and BL datasets, it is not severe, as can be seen from the high rates of the count agreement and low |*DiC*|. Specifically, the |*DiC*| is lower than one on all the datasets, indicating that on average at most one leaf is missed. Due to the occlusion problem, however, the current algorithmic framework is limited to handle 1–2 dozens of leaves at most. Beyond this, occlusion becomes dominant, as no single viewpoint exists from which all the leaves can be seen. For handling mature plants with many dozens of leaves, a single top viewpoint is not enough. Instead, multiple views including side views should be used, and registration of the cameras is required to avoid double counting of leaves across the viewpoints.

The models were trained using datasets containing a single plant in each image. They do not include mechanisms for handling the detection of multiple plants and the assignment of leaves to the correct plant. However, when there are several plants in a single image, a simple solution may incorporate an object detector as the first stage to detect each of the plants, and then count leaves in each detected bounding box independently. Such a two-stage method was applied successfully in Khoroshevsky et al. (2020) for counting wheat spikelets in a spike and bananas in a bunch.

The models suggested here were trained on the LCC and BL datasets, which are limited in viewpoint, illumination conditions, and leaf shape type. If one of these conditions is changed, for example having images taken outdoors rather than in a plantation illumination, re-training of the models would be required to cope with it. While re-training is required, it is likely that the architecture details will not require significant changes. To enable easier re-training, and in some cases even avoid it completely, domain adaptation methods as suggested by Giuffrida et al. (2019) can be utilized.

## 5. Conclusions

The two proposed counting methods improve upon the state of the art results on the test set of the LCC. The better strategy depends on the dataset size. For smaller datasets, multiple-scale regression has a slight advantage, but for a larger dataset with several hundred images the detection-based method is preferable. From an annotation effort point of view, the detection-based approach is more demanding due to its reliance on leaf dot annotation. On the other hand, it provides leaf detection capabilities in addition to counting.

For the detection-based pipe, the most important contributing element is the fusion of the detection results with a regression component. Without it, this approach is inferior in all indices. For the multiple-scale regression approach, an FPN-based representation is helpful, but fusion must be done carefully using confidential information. An interesting and non-trivial direction may be to try and merge the advantages of both methods in a single architecture, providing detection with regression at multiple scales.

Interesting possible extensions of this study may be adapting it to specific agricultural tasks, like in-field leaf counting of weed, or growth-rate estimation in a plantation. In addition, while the suggested methods were developed for leaf counting they can be extended to other counting tasks, like counting flowers or fruits on a tree.

## Data Availability Statement

The datasets presented in this article are not readily available because our banana leaf dataset is the property of a commercial company (“Rahan”) and cannot be made public. The LCC datasets are publicly available. Requests to access the datasets should be directed to guyfar@post.bgu.ac.il.

## Author Contributions

GF and YI developed the main components of the detection-based and regression-based algorithms, respectively, with the guidance of AB-H. FK contributed to the algorithmic developments and experiments. GF prepared the manuscript with AB-H. All authors read and approved the final manuscript.

## Conflict of Interest

The authors declare that the research was conducted in the absence of any commercial or financial relationships that could be construed as a potential conflict of interest.

## Footnotes

^1^https://competitions.codalab.org/competitions/18405.
